# Characteristics of the population eligible for and receiving publicly funded bariatric surgery in Canada

**DOI:** 10.1186/1475-9276-11-54

**Published:** 2012-09-18

**Authors:** Raj S Padwal, Hsui-Ju Chang, Scott Klarenbach, Arya M Sharma, Sumit R Majumdar

**Affiliations:** 1 Department of Medicine, 2F1.26 Walter C. Mackenzie Health Sciences Centre, University of Alberta, 8440-112th Street, Edmonton, T6G 2B7, Alberta, Canada; 2 Alberta Diabetes Institute, Edmonton, Alberta, Canada

**Keywords:** Canada, Bariatric surgery, Health services research, Population health, Access

## Abstract

**Background:**

Bariatric surgery is the most effective current treatment for severe obesity. Capacity to perform surgery within Canada’s public health system is limited and potential candidates face protracted wait times. A better understanding of the gaps between demand for surgery and the capacity to provide it is required. The purpose of this study was to quantify and characterize the bariatric surgery-eligible population in Canada in comparison to surgery-ineligible subjects and surgical recipients.

**Methods:**

Data from adult (age > 20) respondents of the 2007–09 nationally representative Canadian Health Measures Survey (CHMS) were analyzed to estimate the prevalence and characteristics of the surgery-eligible and ineligible populations. Federally mandated administrative healthcare data (2007–08) were used to characterize surgical recipients.

**Results:**

In 2007–09, an estimated 1.5 million obese Canadian adults met eligibility criteria for bariatric surgery. 19.2 million were surgery-ineligible (3.4 million obese and 15.8 million non-obese). Surgery-eligible Canadians had a mean BMI of 40.1 kg/m^2^ (95% CI 39.3 to 40.9 kg/m^2^) and, compared to the surgery-ineligible obese population, were more likely to be female (62 vs. 44%), 40–59 years old (55 vs. 48%), less educated (43 vs. 35%), in the lowest socioeconomic tertile (41 vs. 34%), and inactive (73 vs. 59%). Self-rated mental health and quality of life were lower and comorbidity was higher in surgery-eligible respondents compared with the ineligible populations. The annual proportion of Canadians eligible for surgery that actually underwent a publicly funded bariatric surgery between 2007–09 was 0.1%. Surgical recipients (n = 847) had a mean age of 43.6 years (SD 11.1) and 82% were female. With the exception of type 2 diabetes, obesity-related comorbidity prevalence was much lower in surgical recipients compared to those eligible for surgery.

**Conclusions:**

The proportion of bariatric surgery-eligible Canadians that undergo publicly funded bariatric surgery is very low. There are notable differences in sociodemographic profiles and prevalence of comorbidities between surgery-eligible subjects and surgical recipients.

## 

Extreme obesity (body mass index or BMI ≥ 35 kg/m^2^) in Canada has increased in prevalence by over 400% in three decades and currently afflicts nearly 9% of the adult population [1,2]. Currently, bariatric surgery is widely considered to be the most effective treatment for extreme obesity and contemporary Canadian obesity management guidelines recommend that bariatric procedures be considered in patients who are refractory to non-surgical interventions and who have either severe obesity (body mass index [BMI] ≥ 40 kg/m^2^) or medically complicated moderate obesity (BMI ≥ 35–39.9 kg/m^2^ with a major obesity-related comorbidity such as diabetes or obstructive sleep apnea) [3,4]. Surgery markedly reduces obesity-related morbidity and mortality, improves health-related quality of life, and is cost-effective; in Canada, the incremental cost-effectiveness ratios over a lifetime horizon are $9000-12,000 per quality-adjusted-life-year (QALY) gained [4-7].

From 1996 to 2008, the number of publicly funded bariatric surgeries performed annually in Canada increased 12-fold to 1882 procedures [8,9]. Despite this increase, patients seeking to undergo a publicly funded bariatric procedure in Canada currently face protracted multiyear wait times that average 5 years [10]. Furthermore, residents of six of the thirteen Canadian provinces or territories do not have access to a bariatric surgery program within their home province or territory [9]. These patients must either travel to another Canadian province for publicly funded surgery or pay out-of-pocket to undergo a privately funded procedure.

High demand, driven primarily by the large number of potentially eligible surgical candidates, is thought to be a major contributor to these lengthy wait times [11]. However, no previous study has formally examined the prevalence of the bariatric surgery-eligible population in Canada and calculated the proportion of patients eligible that *actually* receive surgery. Furthermore, no prior study has characterized the surgery eligible Canadian population in comparison to the population considered surgery-ineligible and the population currently receiving bariatric surgery. These data would help inform the current state of bariatric care in Canada, facilitate comparisons with other countries, and assist future health care delivery planning. For example, those responsible for setting volume targets for bariatric surgery in Canada can use these data to understand the current surgical volumes and estimate remaining gaps. Comparing similar data from other countries would allow one to rank surgical volumes in Canada against other health care systems and determine if they require adjustment. To address these knowledge gaps, we linked several contemporary and nationally representative surveys and administrative databases to characterize the population eligible for bariatric surgery in Canada and compare them with the patients currently receiving bariatric procedures.

## Methods

### Characterizing the surgery-eligible population

#### The Canadian health measures survey

Data from the Canadian Health Measures Survey (CHMS; Cycle 1) were analyzed to identify subjects meeting guideline-concordant eligibility criteria for bariatric surgery [4]. The CHMS was a population-representative, cross-sectional survey of 5610 community-dwelling Canadians (aged 6 to 79 years) conducted between 2007–2009 from 15 sites across 5 provinces [12]. Data were collected via in-person interviews conducted in the respondents’ household and physical measures assessments were performed in mobile clinics. Residents of remote regions, Armed Forces personnel, institutionalized persons and individuals residing on Indian Reserves or Crown lands (collectively representing ~3% of the Canadian population) were excluded.

#### Sampling methods

A complex, stratified sampling design was used to obtain estimates representative of the Canadian population [13]. The Canadian Labour Force Survey area frame was used to identify 257 potential survey sites across Canada. A systematic sampling method was then used to randomly select 15 collection sites, with a probability of selection proportional to regional population size. Each site collected data on approximately 350 respondents. Respondents were selected using a two-step process. First, using a selection process stratified by population size and urban proximity, dwellings within 100 km of each collection site were randomly chosen and 1–2 individuals from each dwelling were then randomly selected to participate. Residents of each selected household were informed by mail that an interviewer would visit to collect survey data [13]. Seventy percent of the households randomly selected to participate contributed data.

#### Physical measures and activity assessment

Blood pressure (BP) was measured electronically with calibrated BpTRU automated oscillometric devices (BpTRU Medical Devices Ltd., Coquitlam, British Columbia) using previously described methods [14]. Certified kinesiologists performed anthropometric measurements and supervised fitness assessments. Height was measured using a ProScale M150 digital stadiometer, (Accurate Technology Inc., Fletcher, USA) and weight was measured using a Panther Plus terminal scale (Mettler Toledo Canada, Mississauga, Canada). Obesity was classified according to body mass index (BMI), calculated by dividing the weight in kilograms by the square of the height in meters. Total daily energy expenditure was derived from self-reported three-month activity levels and used to derive a physical activity index (active, moderately active, inactive) for each respondent as previously detailed [15].

#### Definition of comorbidities and health status

The diagnosis of asthma, osteoporosis, dyslipidemia, and mood disorders (including depression, bipolar disorder, mania, anxiety, or dysthymia) was based upon self-report. Respondents were considered hypertensive if they self reported hypertension, were receiving treatment with antihypertensive medication or if the average of five their screening blood pressure measurements were ≥ 140 mm Hg systolic or ≥ 90 mm Hg diastolic. Respondents were considered diabetic if they self reported diabetes, were taking diabetes medications or if their fasting blood glucose level was ≥ 7.0 mmol/L. Blood pressure thresholds to diagnose hypertension in patients with diabetes were systolic BP levels ≥ 130 mm Hg or diastolic BP levels ≥ 80 mm Hg. Last, subjects were asked to rate their mental health and their quality of life as excellent, very good, good, fair or poor [15].

#### Defining eligibility for bariatric surgery

We examined patients aged 20–60 years who, in accordance with Canadian bariatric guidelines, had a BMI ≥ 40 kg/m^2^or a BMI of 35–39.9 kg/m^2^ and a major obesity-related comorbidity (hypertension, diabetes, dyslipidemia and osteoarthritis) and, therefore eligible for bariatric surgery (n = 2850) [4]. Subjects not meeting either of these criteria were categorized as ineligible for bariatric surgery and were sub-categorized into obese (BMI 30–34.9 kg/m^2^), overweight (BMI 25–29.9 kg/m^2^) and normal/underweight (BMI ≤ 24.9 kg/m^2^) categories.

### Characterizing the population receiving bariatric surgery in Canada

The Canadian Institute for Health Information Discharge Abstract Database (CIHI-DAD) and the National Ambulatory Care Reporting System (NACRS) were used to identify patients who received publicly funded bariatric surgery at a Canadian hospital in fiscal 2007–08 (i.e., April 1, 2007 to March 31, 2008). The CIHI-DAD contains acute-care hospital discharge data for all Canadian provinces excluding Quebec, while NACRS contains information on day surgeries performed across Canada (excluding Quebec and Alberta) [16,17]. CIHI-DAD and NACRS data regarding bariatric procedures was available for all provinces except Quebec. Since bariatric procedures are not performed as day surgeries in Alberta, complete data were available for all Canadian provinces and territories except Quebec. The International Statistical Classification of Diseases and Related Health Problems, 10th Revision, Canada (ICD-10-CA) coding system is used to classify medical diagnoses and the Canadian Classification of Health Interventions (CCI) is used to classify surgical procedures in both databases. The frequency of missing data within the CIHI-DAD and NACRS is less than 0.5% [16,17]. In a recent validation study, surgical procedure codes within the CIHI-DAD were 93% accurate compared with chart abstraction [16,17].

Patients undergoing bariatric surgery were identified using the CCI code for bariatric procedures (1.NF.78.^^). Patient age and sex, the type of bariatric procedure, obesity-related co-morbidities, length of stay, in-hospital mortality and in-hospital complications were obtained for 2007–08 fiscal year to coincide and overlap with administration of the CMHS to the Canadian population.

### Statistical analysis

Analyses were primarily descriptive in nature, consisting of estimates of means and proportions. For CHMS data, population-representative estimates were obtained by applying respondent-specific survey weights [15] and bootstrap techniques were used to estimate 95% confidence intervals [18]. Subjects with missing values for a given CHMS survey question were excluded from the analysis of that question. Missing values were present in 4% of responses for household income, 3% of responses for osteoarthritis and less than 1% for all other variables.

To estimate the annual proportion of eligible patients actually receiving bariatric surgery between 2007–09, we divided 1695 (the average annual number of bariatric procedures performed in Canada between 2007–09) by the surgery-eligible population estimate derived from CHMS data [9]. We obtained this figure from previously published Statistics Canada estimates of Canadian bariatric procedure volumes between 2007–09 and used it in preference to our CIHI data estimates because our data did not include the province of Quebec, whereas these Statistics Canada estimates did [9]. All analyses were conducting using SAS (Version 9; Cary, NC) and SUDAAN (Version 10). Ethics approval to conduct this analysis was obtained from the University of Alberta Health Research Ethics Board.

## Results

### Characterizing the surgery-eligible population

**Figure 1:**
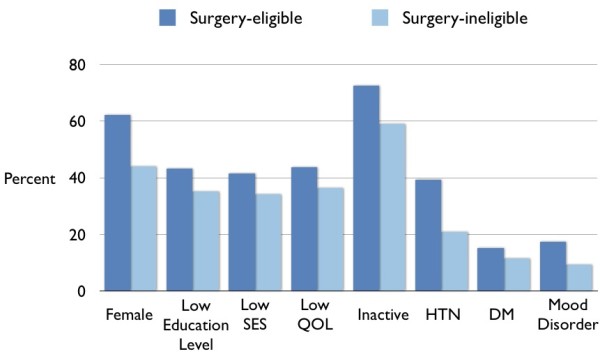
**Characteristics of obese Canadians (BMI ≥ 30 kg/m**^**2**^**) considered eligible and ineligible for bariatric surgery.***SES* socioeconomic status; *QOL* quality of life; *HTN* hypertension; *DM* diabetes.

In 2007–09, over 1.5 million Canadian adults met eligibility criteria for bariatric surgery compared to 19.2 million who did not (Table 1). Of those not meeting criteria, 15.8 million were non-obese and 3.4 million were obese. Surgery-eligible Canadians had a mean BMI of 40.1 kg/m^2^ (95% CI 39.3 to 40.9 kg/m^2^) and, compared to the surgery-ineligible obese population, were more likely to be female (62 vs. 44%), 40–59 years old (55 vs. 48%), less educated (43 vs. 35%), in the lowest socioeconomic tertile (41 vs. 34%), and inactive (73 vs. 59%). Mean BMI levels were 40.1 kg/m^2^ for surgery-eligible population (95% CI 39.3 to 40.9) and 32.7 (95% CI 25.7 to 26.5 kg/m^2^) for the obese surgery-ineligible population. Surgery-eligible Canadians were more likely than obese non-eligible subjects to be female (62 vs. 44%), aged 40–59 years old (55 vs. 48%), less educated (43 vs. 35%), in the lowest socioeconomic tertile (41 vs. 34%), and physically inactive (73 vs. 59%) (Table 1 and Figure 1). Self-rated mental health and quality of life were lower in surgery-eligible respondents compared with the rest of the populace (Table 1). Obesity-related comorbidities and mean number of prescriptions were more prevalent in surgery-eligible subjects, including higher proportions of hypertension, diabetes and mood disorders (Table 1). We estimate that 0.1% of surgery-eligible patients received publicly funded bariatric surgery in Canada (including Quebec) in 2007–09.

**Table 1:** Characteristics of Canadians aged 20–60 years considered surgery-eligible and surgery-ineligible according to current guidelines

**Variable**	**Bariatric eligible**	**Ineligible**		
	**(95% CI)**	**(95% CI)**		
		**Obese**	**Overweight**	**Normal/Underweight**
Number of Observations	233	474	1071	1072
Population-projected sample size (rounded to the nearest 100)	1 515 300	3 419 200	7 623 900	8 153 100
Mean BMI (kg/m^2^)	40.1 (39.3-40.9)	32.7 (32.4-32.9)	27.4 (27.3-27.6)	22.2 (22.0-22.4)
Sex (%)				
Male	37.9 (29.3-47.3)	56.1 (50.3-61.7)	60.7 (57.0-64.2)	39.8 (36.3-43.4)
Female	62.1 (52.7-70.7)	43.9 (38.3-49.7)	39.3 (35.8-43.0)	60.2 (56.6-63.7)
Mean Age (years)	45.8 (43.6-48.0)	44.1 (43.0-45.3)	43.6 (42.6-44.6)	39.1 (38.3-39.8)
Age (%)				
20-39	30.0 (24.0-36.9)	37.9 (33.5-42.5)	35.4 (31.7-39.4)	53.6 (50.9-56.3)
40-59	55.0 (47.8-62.1)	48.2 (42.4-54.2)	53.0 (50.0-56.1)	39.8 (37.2-42.5)
60+	15.0 (8.8-24.2)*	13.9 (10.5-18.1)	11.5 (9.5-13.9)	6.6 (4.9-8.8)
Ethnicity (%)				
White	87.1 (77.7-92.9)	86.5 (71.5-94.3)	81.2 (70.3-88.7)	77.7 (66.5-86.0)
Others	12.9 (7.1-22.3)*	13.5 (5.7-28.5)*	18.8 (11.3-29.7)	22.3 (14.0-33.5)*
Highest education level (%)				
Secondary school graduate or less	43.1 (33.3-53.6)	35.0 (29.7-40.7)	27.4 (20.4-35.8)	23.5 (15.8-33.6)*
Some post-secondary education or post-secondary degree	56.9 (46.4-66.7)	65.0 (59.3-70.3)	72.6 (64.2-79.6)	76.5 (66.4-84.2)
Household income (%)				
< $50,000	41.4 (28.7-55.4)	34.2 (26.2-43.2)	25.3 (20.0-31.5)	31.2 (24.5-38.7)
$50,000 - $99,999	38.5 (30.2-47.6)	39.4 (32.0-44.1)	41.2 (35.0-47.7)	37.9 (32.0 (44.1)
$100,000 or greater	20.1 (12.5-30.7)*	26.4 (18.4-36.4)	33.4 (26.2-41.5)	31.0 (27.1-35.2)
Physical activity (%)				
Highly active	11.1 (5.7-20.5)*	20.0 (13.6-28.3)*	20.7 (14.5-28.6)	22.2 (17.6-27.5)
Moderate active	16.4 (10.5-24.6)*	21.0 (14.0-30.3)*	27.0 (23.6-30.6)	24.1 (20.2-28.6)
Inactive	72.6 (63.8-79.9)	59.0 (46.4-70.5)	52.4 (42.9-61.7)	53.7 (46.8-60.4)
Smoking status (%)				
Never	45.9 (34.3-58.0)	44.8 (37.6-52.2)	47.7 (42.2-53.3)	51.2 (46.9-55.5)
Current	20.7 (12.4-32.5)*	19.8 (15.9-24.3)	21.1 (17.3-25.4)	26.8 (21.1-33.4)
Former	33.4 (24.9-43.2)	35.5 (28.6-42.9)	31.2 (28.3-34.2)	22.0 (18.1-26.5)
Self-rated mental health (%)				
Excellent or very good	65.7 (57.7-73.0)	73.3 (66.3-79.3)	78.4 (74.3-82.1)	70.0 (66.0-73.8)
Good, fair or poor	34.3 (27.0-42.3)	26.7 (20.7-33.7)	21.6 (17.9-25.7)	30.0 (26.2-34.0)
Self-perceived quality of life (%)				
Excellent or very good	56.2 (46.6-65.4)	63.8 (54.0-72.7)	70.9 (66.4-75.0)	77.3 (74.7-79.8)
Good, fair or poor	43.8 (34.6-53.5)	36.2 (27.3-46.0)	29.1 (25.0-33.6)	22.7 (20.2-25.3)
Hypertension (%)	39.2 (33.7-45.0)	20.7 (16.2-26.0)	12.6 (9.7-16.2)	6.8 (4.6-10.0)*
Diabetes (%)	15.1 (8.1-26.2)*	11.4 (6.6-19.0)*	7.0 (2.8-16.8)*	3.3 (1.8-6.1)*
Dyslipidemia (%)	63.1 (51.8-73.2)	54.2 (44.6-64.5)	45.7 (40.0-51.9)	25.4 (20.5-31.0)
Mood disorder (%)	17.4 (12.0-24.5)	9.2 (5.3-15.5)*	10.0 (7.6-13.1)	8.2 (5.9-11.3)
Osteoarthritis (%)	19.2 (11.7-29.9)*	7.3 (5.4-9.8)	5.4 (3.6-8.2)*	3.7 (2.0-6.5)*
Asthma (%)	9.9 (6.4-14.8)*	8.4 (5.7-12.3)*	6.4 (4.0-10.1)*	7.3 (5.4-9.7)
Mean number of prescriptions	2.3 (1.9-2.7)	1.7 (1.4-2.1)	1.3 (1.1-1.5)	0.9 (0.8-1.1)

*estimate has a high variability and should be interpreted with caution.

### Characteristics of the population receiving bariatric surgery in Canada

Eight hundred and forty-seven surgeries were performed in Canada (excluding Quebec) from April 1, 2007 to March 31, 2008 (Table 2). The mean age was 43.6 years (SD 11.1) and 82% of surgical recipients were female. The mean length-of-stay was 4.8 days, there were relatively few in-hospital complications, and the in-hospital mortality rate was <0.6% (Table 2). The prevalence of obesity-related comorbidities was generally lower than in the surgery-eligible population (Tables 1 and 2), with the exception of type 2 diabetes. For example, the prevalence of dyslipidemia was 2% (vs. 63% in the surgery eligible) and hypertension, 13% (vs. 39% in the surgery eligible). In contrast, the prevalence of type 2 diabetes was 21% in the surgical recipients versus 15% in the surgery eligible population.

**Table 2:** Characteristics of patients undergoing bariatric surgery in Canada

**Variable**	Fiscal 2007-08*
	**(n = 847)**
**Baseline**	**Mean (SD)**
Age (years)	43.6 (11.1)
	**No. (%)**
Female	696 (82)
Hypertension	111 (13.1)
Dyslipidemia	20 (2.4)
Diabetes	179 (21.1)
Coronary artery disease	8 (0.9)
Cerebrovascular disease	0 (0)
Depression	25 (3.0)
Hypothyroidism	21 (2.5)
Sleep apnea	92 (10.9)
Gastroesophageal reflux	43 (5.1)
Osteoarthritis	11 (1.3)
Cholelithiasis	19 (2.2)
**Post-operative**	**Mean (SD)**
Length-of-stay (days)	4.8 (13.5)
	**No. (%)**
In-hospital mortality	≤ 5 (<0.6)**
Deep venous thrombosis or pulmonary embolism	≤ 5 (<0.6)**
Post-operative myocardial infarction	≤ 5 (<0.6)**
Post-operative stroke	0 (0)
Post-operative respiratory failure	7 (0.8)

*excludes Quebec.

**CIHI data disclosure rules prohibit publication of exact values for small cell counts.

## Discussion

This analysis of nationally representative Canadian data quantifies and characterizes those who are eligible, ineligible and receiving bariatric surgery in this country. Our first major finding is that a very low proportion of bariatric surgery-eligible Canadians is currently receiving surgery annually. The large gap between potential demand and delivered supply is likely a major contributor to protracted wait times for bariatric procedures [10]. The second major finding of this study is that notable differences in sociodemographic profiles and comorbidities are present amongst individuals that are eligible for compared with those actually receiving bariatric surgery in Canada. Most notably, surgery-eligible individuals exhibit poorer self-reported health status and greater burden-of-illness compared to those considered surgery-ineligible. Conversely, surgical recipients are younger and appear to have a very low burden of comorbidity (with the exception of diabetes), suggesting that it is ’lower risk’ patients are more likely to receive these procedures.

In 2006, 0.4% of the surgery-eligible population underwent a bariatric procedure in the US [19]. This proportion is 400% higher than our estimates for Canada and may be partially (but not fully) explained by the 40% higher prevalence of extreme obesity in the US [1]. Most bariatric procedures in the US are performed within the private health care sector [20]. Privately performed adjustable gastric banding is available in 4 Canadian provinces, but these surgeries are not captured within CIHI databases and thus the total number of bariatric procedures (public plus private) performed within Canada is not known. The results of a recent survey of privately funded bariatric clinics in Canada indicated that private clinics may improve access but provide less comprehensive follow-up care, [21] although the quality and accuracy of these data have been questioned [22].

It is clear that only a small minority of surgery-eligible patients is currently able to access a bariatric procedure. The feasibility of increasing the proportion of surgery-eligible patients that receive a publicly funded bariatric procedure in Canada is uncertain and the optimal proportion of eligible patients that should receive surgery remains undefined. Increasing the provision of surgery is contingent upon the availability of surgeons, multidisciplinary bariatric programs, specialized operating rooms and funding. A recent Health Technology Assessment estimated that increasing the provision of bariatric surgery to 5% of eligible Canadians over 5 years would require nearly 32 000 more publicly-funded bariatric procedures at a cost of nearly 500 million dollars [7]. This estimate does not, however, address the issue of whether or not publicly funded procedures should be reserved for patients with selected characteristics (such as those with greater comorbidity or those that are predicted to incur higher future health care costs). Emerging classification systems that reflect obesity-related comorbidity as opposed to BMI alone may prove useful as a means to stratify potential candidates and reduce the eligible population to a more manageable quantity [23].

Many of the sociodemographic differences seen in our analysis comparing the surgery-eligible and ineligible populations in Canada parallel those reported in the US. Specifically, surgery-eligible individuals in the US are more likely to have lower education levels, income status and self-reported health status [19,24]. In contrast, patients receiving surgery within both Canadian and US programs are predominantly of higher socioeconomic status [19,25]. In the predominantly privately-funded US healthcare system, these inequities are readily explained by individual variation in access to health insurance or ability to pay for surgery. However, within Canada’s publicly funded system (which espouses universal accessibility), no obvious reasons for these socioeconomic disparities in access to bariatric surgery are apparent and this issue requires further examination.

Our results also demonstrate that sex-related disparities exist among bariatric surgery recipients in Canada, whereby women are 4 times more likely to undergo surgery compared to men. This pattern is also similar to that reported in the US, and may be partly explained by the approximately two-fold higher prevalence of severe obesity in females compared to males that is present in both countries [1,19,26]. A recent survey of US bariatric surgeons found that sex was not a significant factor in candidate selection; thus, surgeon preference does not appear to explain these findings [27]. Men may be less likely than women to seek treatment because they may be less aware of the health hazards of extreme obesity. Women may be more likely to seek surgery for body image reasons and it is also possible that sex-related differences in the perceived mental and physical health impact of extreme obesity may explain the higher tendency for women to seek surgery [28].

Obesity-related comorbidities were less common in bariatric surgery recipients compared to the surgery-eligible population. This may be a consequence of candidate preselection, whereby surgeons (or programs) preferentially select healthier patients to undergo surgical intervention or related to known under-coding of chronic stable comorbidities that do not impact surgical risk or length of stay [16]. The lack of a relationship between greater comorbidity burden and receipt of surgery has also been found in other publicly funded bariatric programs. In a tertiary care, publicly funded Norwegian bariatric surgery program, patients undergoing surgery were younger, heavier and a greater proportion had earlier-onset obesity [29]. However, a higher obesity-related comorbidity burden was not present in those receiving surgery [29].

Limitations of our analysis are inherent to the data sources examined and the fact that we compared two different, albeit population-representative, data sources (survey data and administrative data). The CHMS was a voluntary survey and, as with all surveys of this type, the generalizability of the results depends upon the absence of systematic differences between responders and non-responders. Certain obesity-related comorbidities (e.g., sleep apnea) considered to be indications for bariatric surgery were not captured in the CHMS and some comorbidities were self-reported. For these reasons, the number of surgery-eligible subjects may have been underestimated. Three potential limitations of the administrative CIHI data should be noted. First, BMI is not captured within administrative databases; therefore, we assumed but could not directly verify that surgical programs are following current guideline-concordant eligibility criteria when selecting surgical candidates. Published data from the largest publicly funded Canadian bariatric programs suggest that this assumption is justified [30-32]. Second, data from Quebec, which accounts for approximately 40% of publicly funded bariatric procedures in Canada, were not available for the analysis of bariatric surgery recipients and this limits the generalizability of our results to this province. However, published data from the two largest Quebec programs suggest that the demographic characteristics of surgical recipients are similar to our reported results [30,32]. Third, as discussed above, administrative data may not fully capture comorbidity prevalence and surgical complication rates resulting in the underestimation of these outcomes. Fourth, we lacked estimates of the number of Canadians undergoing out-of-country bariatric procedures, which are not captured by CIHI data.

In conclusion, our results quantify and characterize the bariatric eligible population in Canada in relation those considered ineligible and those receiving surgery. These data are useful to depict the current state of bariatric surgery delivery in Canada, thus facilitating comparison to other jurisdictions and serving as a benchmark for ongoing and future initiatives related to bariatric health care delivery in this country. Our findings raise a number of questions regarding the adequacy and appropriateness of publicly funded bariatric surgery care delivery in Canada. In this regard, we note that four of the five founding principles of the Canada Health Act (which outlines the objectives of publicly funded health care delivery in this country) are not currently being met. These principles are comprehensiveness (i.e., all services deemed essential are provided), public administration (obviating the need for private service delivery), universality (all residents receive equal care), accessibility (in a timely manner), and portability (across all provinces and territories). We propose that future efforts should focus on fully characterizing these care gaps; clarifying the role of privately delivered care; examining the value of prioritization methods to streamline and optimize care; and ensuring accessibility for suitable candidates seeking bariatric surgery.

## Competing interests

RP, SWK, SM and AMS are supported by an alternative funding plan from the Government of Alberta and the University of Alberta. AMS is supported by an Alberta Health Services Chair in Obesity Research and Management. SWK and SM hold salary support awards from Alberta Heritage Foundation for Medical Research and Alberta Innovates-Health Solutions. The authors declare no competing interest with respect to this work.

## Authors’ contributions

RP developed the initial study concept, which was refined after input from all of the other authors. HC and RP performed data analysis. RP wrote the initial draft, which was critically revised by all other authors. All authors read and approved the final manuscript.
